# Reduced GIRK expression in midbrain dopamine neurons during prolonged abstinence from fentanyl self-administration

**DOI:** 10.1007/s00213-025-06747-5

**Published:** 2025-02-03

**Authors:** Narges Pachenari, Amy L. Channell, Andrew J. Belilos, Samuel J. Dienel, Khaled Moussawi

**Affiliations:** 1 Department of Psychiatry, University of Pittsburgh, Pittsburgh, PA USA; 2 Department of Neurobiology, University of Pittsburgh, Pittsburgh, PA USA; 3 Intramural Research Program, National Institute On Drug Abuse, Baltimore, MD USA; 4 Department of Neurology, University of California San Francisco, San Francisco, CA USA

**Keywords:** Fentanyl, Dopamine, Ventral tegmental area, GIRK channels, GABA_B_ receptors, Positive allosteric modulator

## Abstract

**Rationale:**

Despite decades of research and medical development, relapse to drug seeking continues to be a significant challenge in the treatment of substance use disorders. GABA_B_ receptor (GABA_B_-R) agonists have been shown preclinically to inhibit relapse by acting on midbrain dopamine (DA) neurons and are sometimes used off-label for the treatment of alcohol use disorder. Studies in rodent models show reduced GABA_B_-R signaling in DA neurons after exposure to stimulants. Similarly, our recent data demonstrated reduced GABA_B_-R currents in DA neurons during prolonged abstinence from fentanyl vapor self-administration (SA). However, the mechanism of opioid-induced changes in GABA_B_-R currents is not well understood. In addition, GABA_B_-R agonists are plagued with a plethora of side effects limiting their potential clinical use.

**Objectives:**

In this study we aimed to answer the following questions: first, can we use GABA_B_-R positive allosteric modulators (PAMs) to inhibit relapse to opioid seeking? Secondly, how do opioids result in reduced GABA_B_-R signaling during prolonged abstinence?

**Approach:**

To this end, we tested the effects of a novel GABA_B_-R PAM (KK-92A) on reinstatement of drug seeking in a rat model of intravenous (IV) fentanyl SA. Using in situ hybridization with RNAscope, we examined the effects of opioids on mRNA levels of various genes involved in GABA_B_-R signaling, in two rodent models of opioid addiction including a rat model of IV fentanyl SA and a mouse model of fentanyl vapor SA.

**Results:**

Our results show that KK-92A inhibits relapse to fentanyl but not sucrose-seeking in rats, and fentanyl SA results in reduced mRNA levels of the G protein-coupled inwardly rectifying potassium channel subtypes 2 and 3 (GIRK_2/3_).

**Conclusion:**

These findings suggest that PAMs like KK-92A are a potential therapeutic strategy for opioid use disorder and their effect is likely due to rectifying GABA_B_-R mediated inhibition of midbrain DA neurons, which is reduced after opioid SA due to reduced GIRK_2/3_ expression.

**Supplementary Information:**

The online version contains supplementary material available at 10.1007/s00213-025-06747-5.

## Introduction

Opioid use disorder (OUD) is a major public health problem with devastating societal and economic costs. The number of opioid overdose deaths in the US has been steadily increasing over the past two decades (U.S. 2021). OUD is characterized by high relapse rates upon exposure to stressors, contexts, or cues previously associated with drug use, even after prolonged abstinence (Koob 2013, 2020; Volkow et al. 2019; Strang et al. 2020). Current FDA-approved treatments of OUD including methadone, buprenorphine, and naltrexone have limited efficacy (Volkow et al. 2018; Lee et al. 2018; O’Connor et al. 2020). As such, the relapse rate to opioids has not improved over the past 50 years(Sinha 2011; Hunt et al. 1971). Relapse vulnerability is thought to be rooted in long-lasting drug-induced neuroadaptations in the brain circuits of reward and motivation (Kalivas and Volkow 2005; Aston-Jones and Harris 2004). Thus, there is a pressing need to identify novel treatment targets and efficacious treatments to reduce enduring relapse vulnerability in OUD.

The role of midbrain dopamine neurons in opioid reward and reinforcement is well-established (Corre et al. 2018; Fields and Margolis 2015; Galaj and Xi 2021). Opioids acutely increase dopamine neuron firing and release through disinhibition (Jalabert et al. 2011; Georges et al. 2006). Dopamine release is critical for relapse and appears to be sensitized after repeated opioid exposure (Diana et al. 1999; Johnson and Glick 1993; Cadoni and Chiara 1999; Zijlstra et al. 2008). Clinical and preclinical studies of various substance use disorders (SUDs), including OUD, show increased mesolimbic dopamine release in response to drugs or drug-associated cues during abstinence, indicative of a sensitized hyperdopaminergic state (Volkow et al. 2006; Moeller et al. 2012). Possible mechanisms of such a sensitized state include increased intrinsic excitability, increased excitatory inputs, or reduced inhibition of dopamine neurons. We previously showed that opioids result in reduced GABA_B_-receptor (GABA_B_-R) currents in midbrain dopamine neurons during prolonged abstinence from fentanyl self-administration (SA)(Moussawi et al. 2020), which we explore further in this report. GABA_B_-Rs in the ventral tegmental area (VTA) are expressed post-synaptically in dopamine and GABA neurons and pre-synaptically on various afferent terminals (Ciccarelli et al. 2012). Synaptic or extra-synaptic activation of post-synaptic GABA_B_-R activates Gi-coupled to inwardly rectifying potassium (GIRK) channels, which in turn hyperpolarizes the membrane potential and inhibits action potential (AP) firing and dopamine release (Lüscher and Slesinger 2010; Cruz et al. 2004). Studies on guinea pigs showed that after 7 days of abstinence from morphine, evoked GABA_B_-R currents in dopamine neurons are decreased due to reduced GABA release (Bonci and Williams 1996). Of note, GABA_B_-R signaling is also reduced in early abstinence from stimulants (Sharpe et al. 2015; Munoz et al. 2016; Arora et al. 2011). However, it remains unclear what mediates the reduction in GABA_B_-R currents after opioids, which potentially could be due to opioid-induced changes in GABA_B_-R, GIRK channels, or coupling in GABA_B_-R to GIRK, regulated by molecules like Regulator of G protein signaling 2 (RGS_2_).

In addition, preclinical studies indicate that inhibiting VTA dopamine neurons with GABA_B_-R agonists can prevent relapse to opioids (Yoon et al. 2007; Ciano and Everitt 2003; Leite-Morris et al. 2004). Baclofen is a GABA_B_-R agonist widely used in these studies. Baclofen can inhibit opioid self-administration (Xi and Stein 1999), reinstatement (Spano et al. 2007), locomotor sensitization (Leite-Morris et al. 2004), and opioid-induced dopamine release (Fadda et al. 2003). However, clinical studies regarding the efficacy of baclofen in OUD are limited. Many randomized controlled trials (RCTs) have been conducted to test baclofen efficacy in other SUDs. Whereas the initial studies showed mixed results (Beraha et al. 2016; Bschor et al. 2018), more recent RCTs targeted patients with severe alcohol use disorder and showed a strong, positive, and dose-dependent effect of baclofen in preventing relapse (Garbutt et al. 2021). It is important to highlight, however, that baclofen is a direct agonist that non-discriminately activates central and peripheral GABA_B_-Rs, resulting in poorly tolerated and severe side effects (e.g., sedation, altered mental status, weakness, vertigo), especially at higher doses that may be effective for SUD (Garbutt et al. 2021; Rigal et al. 2020). Hence, there is a need for alternative treatment approaches, such as positive allosteric modulators (PAMs), which activate GABA_B_-R but with minimal side effects. Unlike direct agonists that bind to the GABA_B_-R_l_ subunit, GABA_B_-R PAMs bind to the GABA_B_-R_2_ subunit and induce conformational changes that amplify endogenous signaling (Li and Slesinger 2021). GABA_B_-R PAMs do not possess intrinsic agonist activity and thereby preserve the spatiotemporal resolution of GABA transmission and GABA_B_-R activation, resulting in lower incidence and severity of side effects (Burford et al. 2013). Another advantage of PAMs is that they enhance GABA_B_-R activation without inducing desensitization and tolerance (Sturchler et al. 2017). GABA_B_-R PAMs have been studied in models of stimulants and nicotine use disorders and were shown to inhibit drug-related behaviors like SA and relapse, without affecting responses to natural rewards (Li et al. 2017; Maccioni et al. 2021; Halbout et al. 2011; Miguel et al. 2019). To our knowledge, GABA_B_-R PAMs have not been studied in opioid use disorder models.

In this study, we aimed to examine the effect of a novel GABA_B_-R PAM (KK-92A) on drug- and cue-induced reinstatement of drug-seeking in a rat model of intravenous (IV) fentanyl SA. Furthermore, we aimed to explore the mechanism of GABA_B_-R current attenuation during prolonged abstinence by assessing the effects of opioids on mRNA levels of various genes involved in GABA_B_-R signaling, using fluorescent in situ hybridization, in two models of opioid addiction including a rat model of IV fentanyl SA and a mouse model of fentanyl vapor SA.

## Materials and Methods

### Intravenous (IV) self-administration in rats

#### Animals

Male and female adult Long Evans rats (> 10 weeks old) were housed in cages (two to three per cage) on a 12-h/12-h light/dark cycle in a controlled temperature and humidity environment (21°C ± 2°C). Standard rodent chow and water were available in the home cage ad libitum. Experiments took place between 9:00 am and 6:00 pm, 5 days/ week. All experiments were approved by the Institutional Animal Care and Use Committee at the University of Pittsburgh.

#### Self-administration

Rats underwent catheter surgery then were trained to self-administer IV fentanyl (3 µg/kg) or oral sucrose (10%) for 3 h per session on an FR1 schedule as detailed in the Supplementary Materials and Methods.

#### Extinction and reinstatement

After 22 days of fentanyl or sucrose self-administration, a 2-week extinction period began in which lever presses did not result in reward delivery or cue presentation. Extinction sessions lasted 3 h. After the last session of extinction, rats underwent reinstatement testing in 1-h sessions. Cue reinstatement was tested in all animals after vehicle vs. KK-92A (10 mg/kg) treatment intraperitoneally, 1-h prior to the session. In these sessions, an active lever press resulted in cue presentation with no reward delivery. The vehicle vs. KK-92A treatments were counterbalanced. Animals went through 2–3 days of extinction prior to repeat reinstatement testing.

In the fentanyl group, drug-induced reinstatement was also tested in a separate session 2–3 days after the last cue reinstatement session. In these sessions, fentanyl (30 µg/kg, s.c) was injected 5 min before the session. For the sucrose group, only cue-induced reinstatement was performed.

### Vapor self-administration in mice

#### Animals

Adult C57BL/6 mice, both male and female and aged over 10 weeks, were sourced from The Jackson Laboratories. Mice were accommodated in groups of two to four per cage and were kept in conditions with a 12-h/12-h light/dark cycle at a temperature of 21 ± 2°C. All experiments were carried out during the dark cycle. Mice had free access to food and water in their home cages. All experimental procedures were approved by the Policy on Humane Care and Use of Laboratory Animals, University of Pittsburgh.

#### Self-administration

Operant conditioning chambers and set-up are detailed in the Supplementary Materials and Methods. One of the nose poke holes was designated as active, leading to vapor delivery and light cue presentation, while the other nose poke hole remained inactive with no programmed consequences. Active nose pokes triggered vapor delivery for approximately 1 min, accompanied by cue light presentation and a subsequent 1-min timeout period. All nose pokes were recorded throughout the entire session. The sessions had durations of 1 h, following a fixed ratio 1 (FR1) or FR3 schedule of reinforcement where one or three operant responses on the active operandum resulted in 1 vapor delivery, respectively. In the case of vehicle self-administration, only vehicle vapor was delivered in response to active nose pokes, excluding fentanyl. Mice underwent five self-administration sessions per week, with one session per day, for 4 weeks.

#### Drugs

KK-92A (10 mg/kg) was obtained from the chemistry core at the Moffitt Cancer Center (FL). KK-92A was dissolved in a mixture of dimethyl sulfoxide, polysorbate 80, and distilled water (ratio of the mixture components: 5:10:85) as previously described (Li et al. 2017) and administered intraperitoneally 1 h before reinstatement testing. Fentanyl-HCl was obtained through the National Institute on Drug Abuse Drug Supply Program. For IV SA, fentanyl was dissolved in saline (30µg/mL/kg). For vapor SA, fentanyl was dissolved in a mixture of 80% vegetable glycerol and 20% propylene glycol. The process involved dissolving fentanyl at a concentration of 3 mg/ml through interleaved vortexing and sonication (~ 30 min in total), followed by filtration. Gentamicin (4.25 mg/mL/kg) was diluted in saline. Heparin (40 units/mL) was mixed with Baytril (2.27%) and saline to flush IV catheters daily (starting one week after surgery). Carprofen (5 mg/mL/kg) was administered on the day of and one day after surgery to ease pain. Propofol (10 mg/mL) was used to test catheter patency.

### mRNA fluorescent in situ hybridization

#### Tissue preparation

Four weeks after the last behavioral sessions in the experiments above, the brains of mice and rats were extracted and flash-frozen in 2-methyl-butane on dry ice. The brains were then wrapped in aluminum foil, sealed in an airtight container, and stored at −80°C. Subsequently, the brains were sliced (coronally) into 20 µm sections using a Leica Cryostat and collected onto Superfrost Plus™ microscope slides, spanning from Bregma level −4.9 to −6.1 to capture the VTA. These slices were stored in a slide box at −80°C until the *in-situ* hybridization procedure could be conducted. One section per animal at the same rostral/caudal level was used for the next steps.

#### In-situ hybridization

Tissue samples were fixed in 4% PFA and dehydrated in ethanol. RNAscope® v_2_ assay (Advanced Cell Diagnostics, Inc.) was performed according to the manufacturer’s instructions as outlined in their RNAscope® Multiplex Fluorescent Reagent Kit v_2_ Assay protocol for fresh frozen tissue samples. The following probes were used in brain slices: GIRK_2_ (catalog # 828,641), GIRK_3_ (catalog # 578,891), RGS_2_ (catalog # 1,102,351-C4), GABA_B_-R1 (catalog # 546,461-C2), and tyrosine hydroxylase (TH) (catalog # 314,651-C3) in mice brain slides, and GIRK_2_, dopamine receptor subtype 2 (D_2_-R) (catalog # 406,501-C4), GABA_B_-R1, and TH in rats brain slides.

#### Microscopy and image processing

All mRNA analyses were blinded to the treatment group. Samples were imaged using a widefield fluorescent microscope (Olympus VS200 Slideview) and a 40 × objective with extended focal imaging (EFI) around the region of interest (ROI) (midbrain). TH signal was used to identify dopamine neurons in the VTA and substantia nigra pars compacta (SNc) to determine the ROI. Five fluorescent channels were used: DAPI, FITC, Cy3, Cy5, and Cy7. Whole slice images were also acquired at 40 × but without EFI. Exposure times were optimized by ensuring no oversaturation occurred for each of the five fluorescent channels and were kept the same across different imaging sessions.

Data were quantified using QuPath Bioimage Analysis cell counting software (Bankhead et al. 2017). Positive cell detection was run on each image using TH to identify dopamine neurons. We then measured the mean pixel intensity of the other labels within the identified ROIs.

For the mediolateral distribution of mRNA expression (Fig. 2F, G, K, O and Fig. 4F and J), data were binned into 50 µm bins between the midline and lateral SNc. The mean density of GIRK_2/3_ mRNA expression was calculated for TH-positive neurons in each bin.

#### Data analysis

mRNA analysis was blinded. Data are presented as mean ± SEM. Data were tested for normality and appropriate statistical testing was used accordingly using the GraphPad prism software. The specific tests used are described for each experiment. Statistical significance was set at 0.05.

## Results

### The effect of KK-92A on cue- and drug-induced reinstatement after IV fentanyl self-administration in rats

**Fig. 1:**
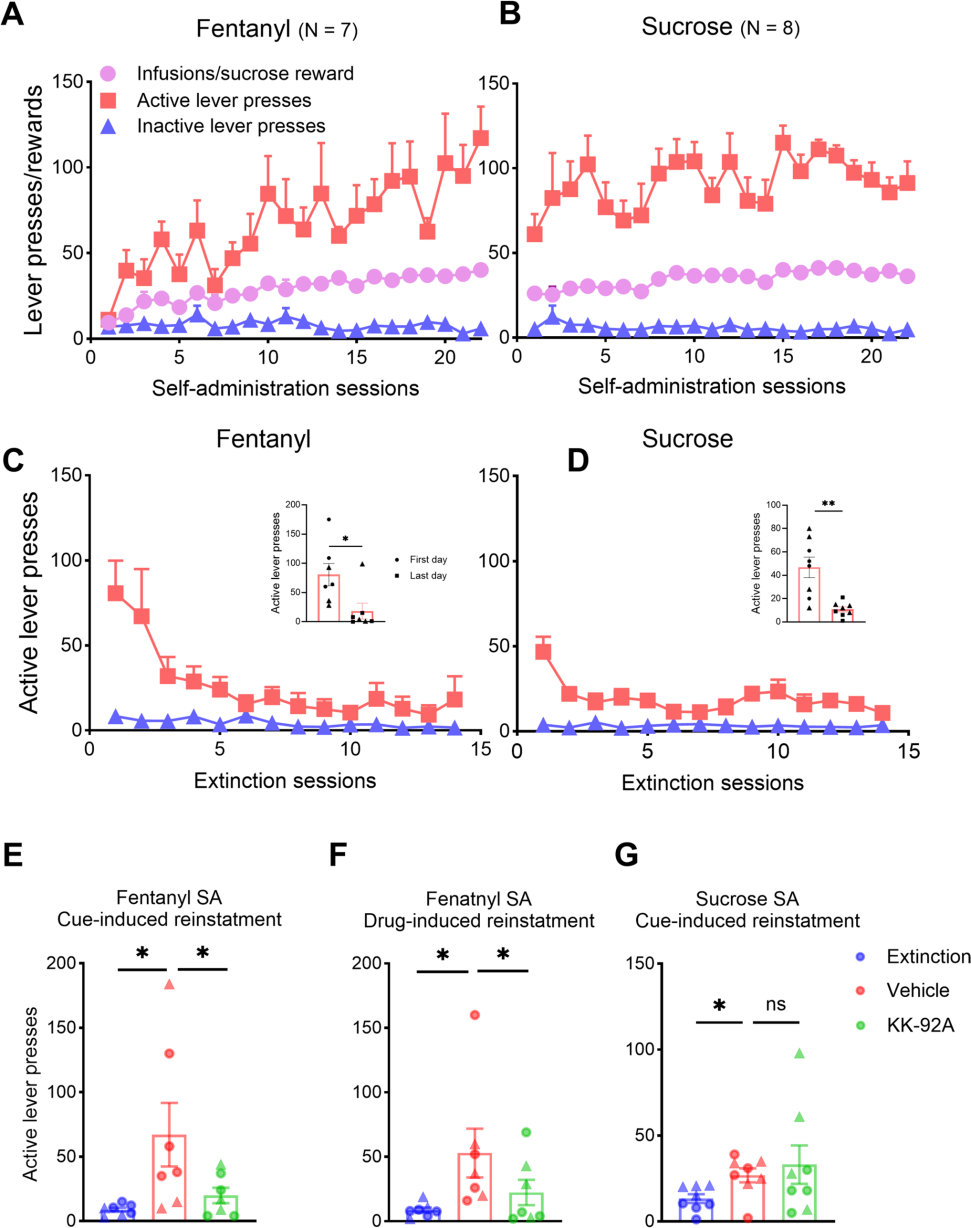
KK-92A inhibits cue- and drug-induced reinstatement of fentanyl seeking. **A** Rats self-administered intravenous fentanyl (3µg/kg/infusion) for 22 days on an FR1 schedule of reinforcement. The number of active and inactive lever presses and reward deliveries are depicted. **B** Another group of rats self-administered sucrose (10%) orally for 22 days on an FR1 schedule of reinforcement. The number of active and inactive lever presses and reward deliveries are depicted. **C** and **D** Animals from both groups underwent extinction training for 2 weeks. The graphs show the number of active and inactive lever presses during this period. The insets show the comparison between the first and the last day of extinction (paired t-test,* t*_6_ = 2.82, *p* = 0.03 for the Fentanyl group and t-test,* t*_7_ = 4.25, *p* = 0.003 for the sucrose group). **E**–**G** Blue bars show the number of active lever presses in the last session of extinction. Animals received a single dose of vehicle (red bars) or KK-92A (green bars) intraperitoneal injections before the reinstatement tests (the order of treatments was counterbalanced). **E** Cue-induced reinstatement of fentanyl self-administration was inhibited with KK-92A but not vehicle treatment (One-way RM ANOVA, *F*_(2, 12)_ = 5, *p* = 0.026; post-hoc multiple comparisons with Holm Sidak test). The number of active lever presses increased significantly on the first day of reinstatement compared to the last day of extinction (*t*_12_ = 3, *p* = 0.023), and it decreased significantly when animals were injected with KK-92A before the reinstatement test (*t*_12_ = 2.4, *p* = 0.032). **F** KK-92A similarly inhibited drug-primed reinstatement with subcutaneous fentanyl (30 µg/kg) (One-way RM ANOVA, *F*_(2, 12)_ = 5.24, *p* = 0.023, post-hoc multiple comparisons with Holm Sidak test). The number of active lever presses increased significantly on the first day of reinstatement in comparison to the last session of extinction (*t*_12_ = 3.16, *p* = 0.016), and it decreased when the animals received KK-92A before the session (*t*_12_ = 2.18, *p* = 0.05). **G** Cue-induced reinstatement of sucrose self-administration was not inhibited with KK-92A (One-way RM ANOVA, *F*_(1.15, 8.11)_ = 2.7, *p* = 0.13, post-hoc multiple comparisons with Holm Sidak test). The number of active lever presses during the first session of reinstatement increased significantly in comparison to the last session of extinction (*t*_7_ = 4.01, *p* = 0.01). However, no differences were observed when the animals received KK-92A before the test session (*t*_7_ = 0.59, *p* = 0.57). Triangle and circle data points in bar graphs represent data from female and male animals, respectively

In this experiment, we aimed to explore whether the activation of GABA_B_-Rs using a positive allosteric modulator (PAM), KK-92A, could inhibit drug-seeking behavior in rats. Rats were trained to lever press for oral sucrose or IV fentanyl over 22 days (Fig. 1A, B). In both groups, rats learned to lever press for reward and most rats reached the maximum number of rewards per session (set at 45). Interestingly, escalation of drug intake (slope alpha = 1.18, R^2^ = 0.86, p < 0.0001) and lever pressing (slope alpha = 7.18, R^2^ = 0.73, p < 0.0001) could be observed in the fentanyl group (Fig. 1A).

After the final training session, the animals underwent extinction training for two weeks (Fig. 1C and D). In both groups, the number of active lever presses on the last day of extinction was significantly lower compared to the first day (Fig. 1C and D insets). Extinction sessions were followed by cue-induced reinstatement. Our results show a significant reinstatement of lever pressing in both fentanyl and sucrose groups with the vehicle treatment but inhibited reinstatement of lever pressing in the fentanyl group with the KK-92A treatment (Fig. 1E and G). Interestingly, KK-92A did not inhibit the reinstatement of lever pressing in the sucrose group compared to vehicle treatment (Fig. 1G), suggesting a selective effect of KK-92A on drug seeking. We also tested the effect of KK-92A on drug-induced reinstatement in the fentanyl group and found similar results to the KK-92A effects on cued reinstatement (Fig. 1F). No differences were observed in the number of inactive lever presses between the two groups (Fig. S1). These data demonstrate that KK-92A inhibits drug-seeking behavior in rats in response to drug cues or drug primes and that this effect is specific to the drug but not sucrose seeking.

### GABA_B_-R related mRNA levels during prolonged abstinence after IV fentanyl self-administration in rats

**Fig. 2:**
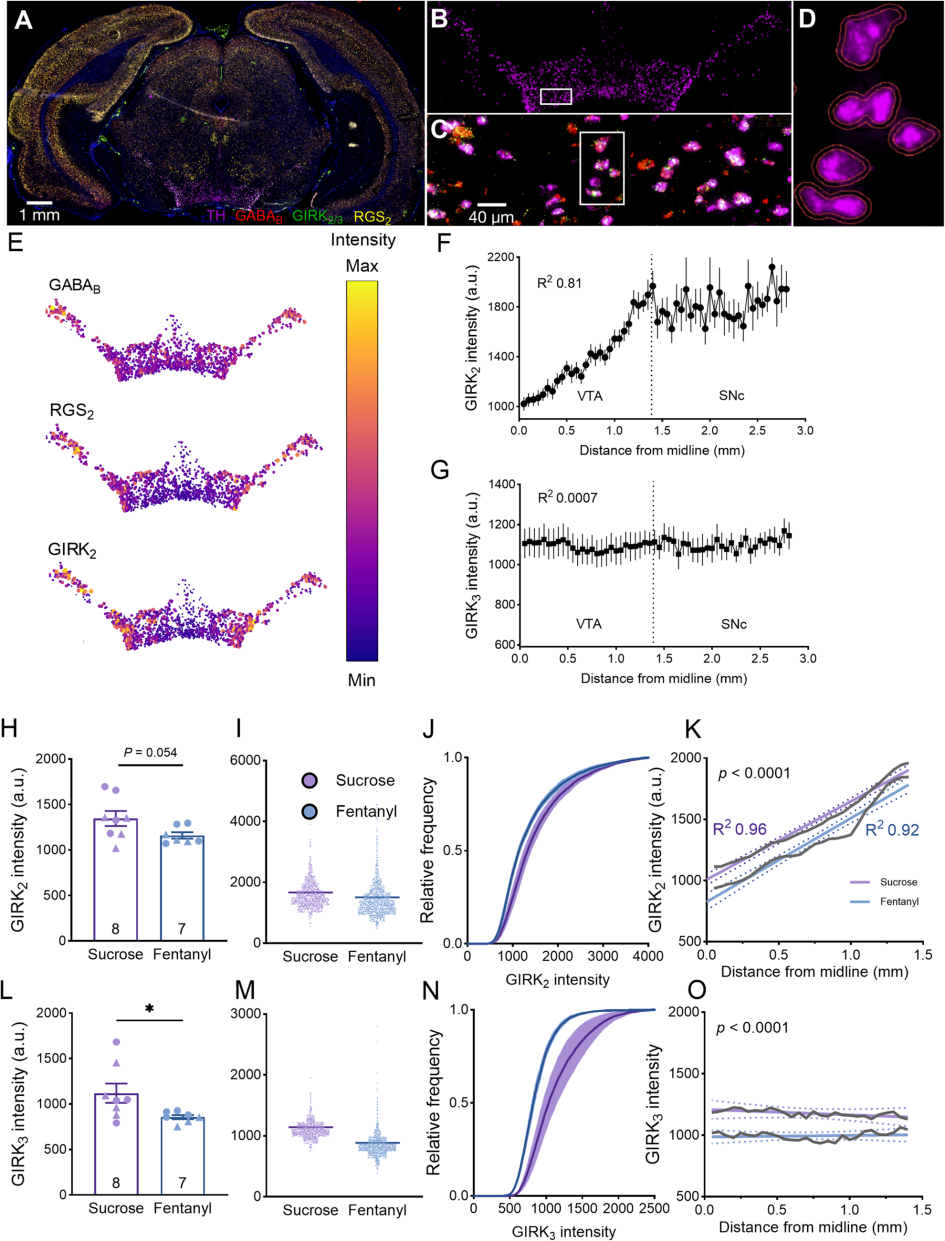
Reduced GIRK_2_ and GRIK_3_ mRNA expression in midbrain dopamine neurons during prolonged abstinence from IV fentanyl self-administration in rats. **A**-**D** In situ hybridization with RNAScope was performed to identify GIRK_2_/GIRK_3_ (green), GABA_B_ (red), RGS_2_ (yellow)_,_ and TH (magenta) mRNA levels in VTA neurons. The blue color corresponds to DAPI staining to mark cell nuclei. **A** A representative image of a rat coronal section with all channels overlaid. **B** TH mRNA expression identifies the midbrain dopamine neurons from A. **C** Zoom-in of inset in B with an overlay of all channels. **D** Zoon-in of inset in C to illustrate an example cell detection based on the TH channel using the QuPath software. **E** Example spatial map of GABA_B_, RGS_2_, and GIRK_2_ mRNA expression in all midbrain dopamine neurons in one coronal slice showing low to high mediolateral expression gradient. Larger circles and brighter colors indicate higher intensity. **F** Quantification of mediolateral GIRK_2_ mRNA expression in VTA and SNc across all animals; note the expression gradient in the VTA that levels off in SNc. **G** Quantification of mediolateral GIRK_3_ mRNA expression across all animals showed no gradient in the VTA and SNc. **H** Mean GIRK_2_ mRNA expression in the VTA. Each data point is the mean of GIRK_2_ intensity in all dopamine neurons in each rat. Data show a strong trend toward reduced GIRK_2_ mRNA in the fentanyl group (Mann Whitney U test, U = 11, *p* = 0.054). **I** A dot plot of data in H but from all neurons in all animals showed reduced intensity in the fentanyl group (mean ± SEM: 1644 ± 10.99 in the sucrose group and 1466 ± 10.48 in the fentanyl group). **J** Cumulative distribution of all neurons/groups for the GIRK_2_ intensity showed a leftward shift in the fentanyl group (Kolmogorov–Smirnov D = 0.0662, *p* < 0.0001). **K** Linear regression of GIRK_2_ intensity along the mediolateral axes of VTA (to account for the gradient of expression) showed lower GIRK_2_ mRNA expression throughout the VTA (similar slopes (*F*_1, 416_ = 0.44, *p* = 0.5) but different intercepts (*F*_1, 417_ = 30.304, *p* < 0.0001)). **L** Mean GIRK_3_ mRNA expression presented like in H showed a significant reduction for GIRK_3_ mRNA in the fentanyl group (Mann Whitney U test, U = 9, *p* = 0.029). **M** A dot plot of all the data points of GIRK_3_ intensity in each group showed a decrease in the Fentanyl group (mean ± SEM: 1132 ± 4.24 in the sucrose group and 875.9 ± 3.12 in the Fentanyl group). **N** Cumulative distribution of GIRK_3_ mRNA expression in all VTA dopamine neurons showed a leftward shift in the fentanyl group (Kolmogorov–Smirnov D = 0.2411, *p* < 0.0001). **O** Linear regression of GIRK_3_ intensity along the mediolateral axis of VTA (to account for the spatial gradient of expression) showed lower GIRK_3_ mRNA expression throughout the VTA (similar slopes (*F*_1, 416_ = 0.85, *p* = 0.35) but different intercepts (*F*_1, 417_ = 54.52, *p* < 0.0001)).a.u. = arbitrary unit. Triangle and circle data points in bar graphs represent data from female and male animals, respectively

To further explore the mechanisms underlying the previously observed reduction in GABA_B_-R currents during prolonged fentanyl abstinence, we utilized mRNA in situ hybridization with RNAScope to assess the mRNA expression levels of GABA_B_-R1 (an obligatory subunit of GABA_B_-R (Qian et al. 2023)), GIRK_2_ and GIRK_3_ (the main GIRK subunits expressed in dopamine neurons (Cruz et al. 2004)), RGS_2_ (a main GABA_B_-R regulator in dopamine neurons (Labouèbe et al. 2007)), and TH. Brain slices were collected approximately four weeks after the conclusion of the behavioral tests and RNAScope was performed. The goal of the 4-week delay is to assess long-lasting changes related to sustained relapse vulnerability, which are not confounded by changes due to acute withdrawal. An example coronal section sample displaying all channels (GIRK_2_, GIRK_3_, GABA_B_, RGS_2_, TH, and DAPI) is presented in Fig. 2A. TH-positive (TH^+^) neurons served to identify midbrain dopamine neurons in the VTA and SNc (Fig. 2B-D). The intensity of the different channels representing the mRNA expression of GIRK_2_, GIRK_3_, GABA_B_-R1, and RGS_2_ was then measured in individual TH^+^ neurons. The spatial maps of GABA_B_-R_1_, RGS_2_, and GIRK_2_ mRNA expression showed a mediolateral gradient as previously described (Lammel et al. 2008; Reyes et al. 2012) (Fig. 2E, F). However, no mediolateral differences were observed in the expression of GIRK_3_ in these regions (Fig. 2G). As the TH mRNA was used for dopamine neuron identification, TH mRNA expression was compared and found to be similar between the fentanyl and sucrose groups (Fig. S2). Importantly, the intensity of GIRK_2_ and GIRK_3_ was reduced in the fentanyl group compared to the sucrose group (Fig. 2H-O). For GIRK_2_, intensity values were averaged across all neurons in each rat (Fig. 2H), which does not factor in the variance of data, especially along the mediolateral axis. Thus, we analyzed the data in both groups while incorporating the spatial distribution of GIRK_2_ expression using linear regression between the distance from the midline (x-axis) and GIRK_2_ intensity (y-axis) (Fig. 2K), which highlighted the difference between the groups. The results revealed no differences in the slope of the fitted line; however, there were significant differences in the intercepts, reflecting a reduction in GIRK_2_ intensity in the fentanyl group throughout the VTA (Fig. 2K). The intensity of GABA_B_-R_1_ and RGS_2_ mRNA were compared, and no differences were observed between the two groups (Fig. S3).

This experiment suggests that GIRK_2_ and GIRK_3_ mRNA expression is reduced in dopaminergic neurons of the midbrain during prolonged abstinence from fentanyl SA, while the mRNA levels of GABA_B_-R1 and RGS_2_ are not affected by fentanyl SA during the same timeframe. In the next step, our aim was to conduct similar experiments in a different fentanyl SA model, namely fentanyl vapor SA in mice that we had previously developed and validated (Moussawi et al. 2020; Marchette et al. 2021), to determine whether these results would generalize across species and models.

### Fentanyl vapor self-administration in mice

**Fig. 3:**
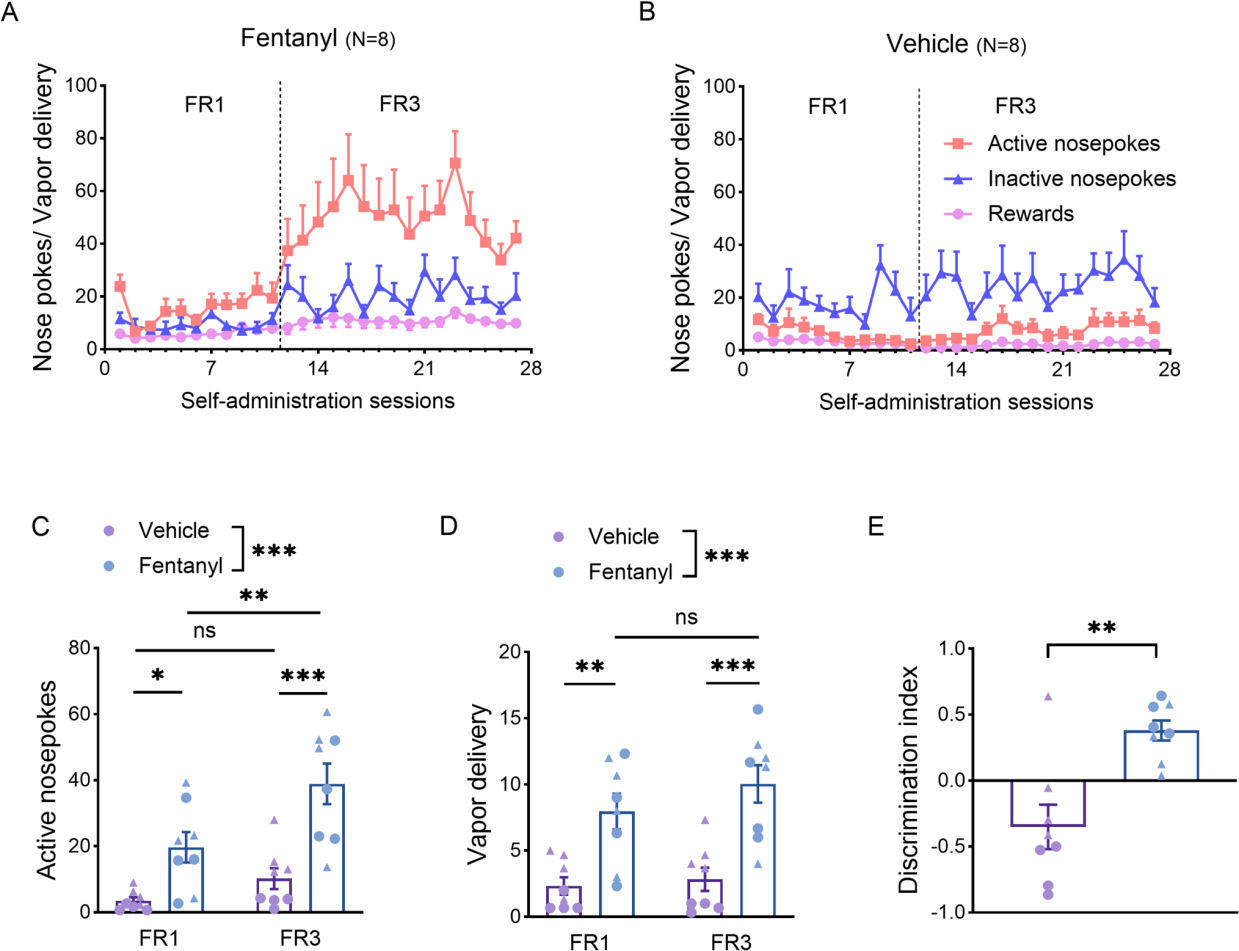
Mice fentanyl vapor self-administration in response to different reinforcement schedules. **A** and **B** Mice self-administered fentanyl (A) or vehicle (B) in 1-h sessions on an FR1 schedule (11 sessions) followed by an FR3 schedule (16 sessions). The graph shows the number of active and inactive nose pokes and vapor deliveries in each session. **C** Data showing the number of active nose pokes for the last 3 sessions of FR1 and FR3 in both groups. Two-way ANOVA showed significant effects for group and schedule of reinforcement (*F*_1, 14_ = 21.22, *p* = 0.0004, post-hoc multiple comparisons with Sidak test). Data show a significantly higher number of active nosepokes in the fentanyl group compared to the vehicle group in FR1 (*t*_28_ = 2.74, *p* = 0.02) and FR3 (*t*_28_ = 4.85, *p* < 0.0001) schedules. Data also show a significantly higher number of active nosepokes in FR3 compared to FR1 schedules in the fentanyl group (*t*_14_ = 4.055, *p* = 0.0024), but not in the vehicle group (*t*_14_ = 1.41, *p* = 0.32). **D** data showing the number of vapor deliveries for the last 3 sessions of FR1 and FR3 schedules in both groups. Two-way ANOVA showed a significant group but not schedule effect (*F*_1, 14_ = 24.41, *p* = 0.0002). Data show a significantly higher number of vapor deliveries in the fentanyl group compared to the vehicle group for both FR1 (*t*_28_ = 3.54, *p* = 0.002) and FR3 (*t*_28_ = 4.53, *p* = 0.0002) schedules. No significant difference was observed in the fentanyl group between FR1 and FR3 schedules (*t*_14_ = 1.6, *p* = 0.24). **E** The discrimination index was calculated in both groups for FR3 schedules. The unpaired *t*-test showed an increase in this index in the fentanyl group in comparison to the vehicle group (*t*_14_ = 3.95, *p* = 0.0014). Triangle and circle data points in bar graphs represent data from female and male animals, respectively

Next, we sought to replicate the GIRK findings in a mouse model of fentanyl vapor self-administration. Both mice and rat models of opioid addiction are used in our study to leverage the complementary strengths of each model, ensure the robustness of our findings, and enhance the translational relevance and generalizability of our conclusions. Two separate groups of mice were trained in 1-h sessions to nose poke for fentanyl or vehicle vapor on an FR1 schedule of reinforcement followed by an FR3 schedule for 16 sessions (Fig. 3A and B). The number of active nose pokes and reward deliveries in the fentanyl group was higher compared to the vehicle group using the FR1 and FR3 schedules (Fig. 3C and D). Mice showed robust fentanyl vapor SA as reflected in the number of vapor deliveries and persistent intake with increased FR compared to vehicle SA, and clear discrimination between active and inactive nose pokes as indicated with a positive discrimination index (Fig. 3E). Moreover, in the fentanyl group, the number of nose pokes in FR3 schedules was significantly higher compared to the FR1 schedule (Fig. 3C) and the number of vapor deliveries remained unchanged (Fig. 3D). In the vehicle group, there was no increase in the number of active nose pokes upon transition from FR1 to FR3 (Fig. 3C).

### GABA_B_-R related mRNA levels during prolonged abstinence after vapor self-administration in mice

After the last session of vapor self-administration, mice were kept in their home cages for 4 weeks to undergo forced abstinence. Then, the brains were harvested, and fluorescent in situ hybridization was performed to measure mRNA expression of TH, GIRK_2_, GABA_B_, and D_2_-R in midbrain dopamine neurons like in the rat experiment above. In this experiment, we elected to forgo GIRK_3_ given its low expression in midbrain dopamine neurons (Burgo et al. 2008) and instead measured D_2_-R mRNA expression given its coupling to the same GIRK channels as GABA_B_-R (Condon et al. 2022).

**Fig. 4:**
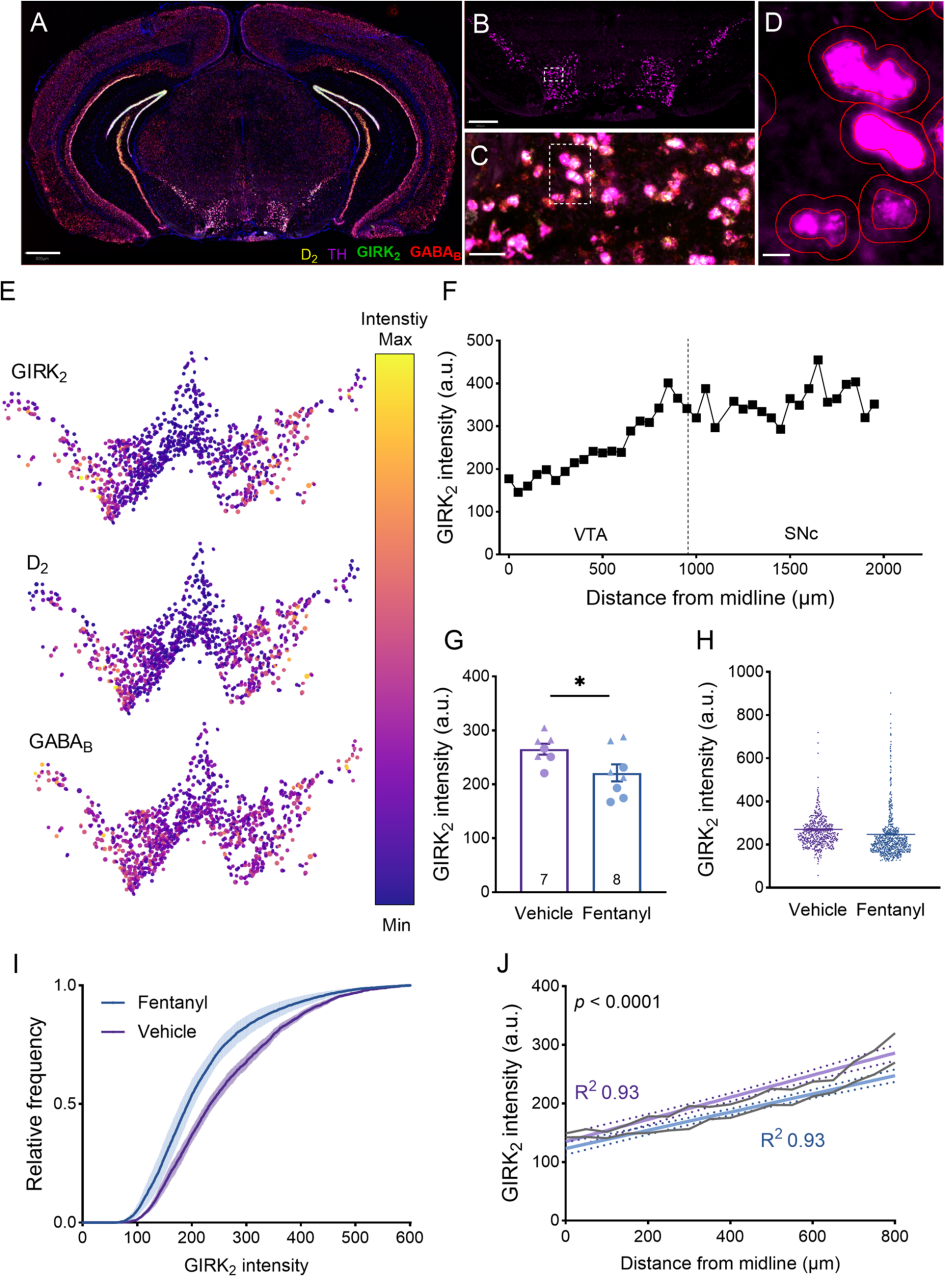
Reduced GIRK_2_ mRNA expression in midbrain dopamine neurons during prolonged abstinence from vapor fentanyl self-administration in mice. **A**-**D** In situ hybridization with RNAScope was performed to identify GIRK_2_ (green), GABA_B_ (red), D_2_ (yellow)_,_ and TH (magenta) mRNA levels in the midbrain. The blue color corresponds to DAPI staining to mark cell nuclei. **A** A representative image of a mouse coronal section with all channels overlaid. Scale bar: 800 µm. **B** TH mRNA expression identifies the midbrain dopamine neurons from A. Scale bar: 400 µm. **C** Zoom-in of inset in B with an overlay of all channels. Scale bar: 50 µm. **D** Zoon-in of inset in C to illustrate an example cell detection based on the TH channel using the QuPath software. Scale bar: 10 µm. **E** Example spatial map of GABA_B_, D_2_, and GIRK_2_ mRNA expression in all midbrain dopamine neurons in one coronal slice showing low to high mediolateral expression gradient. Larger circles and brighter colors indicate higher intensity. **F** Quantification of mediolateral GIRK_2_ mRNA expression in VTA and SNc in one animal; note the expression gradient in the VTA that levels off in SNc like the rat data in Fig. 2. **G** Mean GIRK_2_ mRNA expression in the VTA. Each data point is the mean of GIRK_2_ intensity in all dopamine neurons in each mouse. Data show a significant reduction in GIRK_2_ mRNA in the fentanyl group (unpaired *t*-test, *t*_13_ = 2.25, *p* = 0.041). **I** A dot plot of data in G but from all neurons in all animals showed reduced intensity in the fentanyl group (mean ± SEM: 263.7 ± 2 in the vehicle group and 221.2 ± 1.4 in the Fentanyl group). **I** Cumulative distribution of all neurons/groups for the GIRK_2_ intensity showed a leftward shift in the fentanyl group (Kolmogorov–Smirnov D = 0.121, *p* = 0.0003). **J** Linear regression of GIRK_2_ intensity along the mediolateral axes of VTA (to account for the gradient of expression) showed lower GIRK_2_ mRNA expression throughout the VTA (similar slopes (*F*_1, 30_ = 3.81, *p* = 0.06) but different intercepts (*F*_1, 31_ = 33.08, *p* < 0.0001)). a.u. = arbitrary unit. Triangle and circle data points in bar graphs represent data from female and male animals, respectively

An example coronal section sample displaying all channels (GIRK_2_, GABA_B_, D_2_-R, TH, and DAPI) is presented in Fig. 4A. TH^+^ cells served to identify midbrain dopamine neurons in the VTA and SNc (Fig. 4B-D). Similar to rat data, the spatial maps of GABA_B_-R_1_, D_2_-R, and GIRK_2_ mRNA expression showed a mediolateral gradient (Fig. 4E, F). TH mRNA expression was compared and found to be similar between the fentanyl and vehicle groups (Fig. S4). Aligned with rat data, the intensity of GIRK_2_ was reduced in the fentanyl group compared to the vehicle group (Fig. 4G-I). To factor in the mediolateral distribution of GIRK expression, data in both groups were analyzed using linear regression between distance from midline (x-axis) and GIRK_2_ intensity (y-axis). The results revealed no differences in the slope of the fitted line; however, there were significant differences in the intercepts, reflecting a reduction in GIRK_2_ intensity in the fentanyl group throughout the VTA (Fig. 4J). Analysis of the mRNA intensity levels of GABA_B_ and D_2_ revealed no significant differences between the two groups (Fig. S5).

In this part, consistent with the data obtained from the previous section on rats, it was observed that the mRNA levels of GIRK_2_ were decreased in midbrain dopamine neurons of fentanyl-abstinent mice.

## Discussion

In the current study, we tested the effects of a GABA_B_-R PAM, KK-92A, on cue- and drug-induced reinstatement in a rat model of IV fentanyl self-administration. We observed that KK-92A decreased cue- and drug-induced reinstatement of drug but not sucrose seeking. Moreover, mRNA expression analysis revealed a reduction in GIRK_2_ and GIRK_3_ mRNA expression in rat VTA neurons in the fentanyl group, while there were no differences in RGS_2_ and GABA_B_ mRNA expression between the two groups. To test the generalizability of these results across species and models of fentanyl self-administration, we conducted a similar mRNA expression experiment in a mouse model of fentanyl vapor self-administration. The mice mRNA data showed similar results with reduced GIRK_2_ but not GABA_B_-R1 mRNA levels in the fentanyl compared to the vehicle group.

Our KK-92A finding is in line with previous studies in other models of substance use disorders. In a rat model, KK-92A reduced nicotine self-administration and cue-induced nicotine seeking, without affecting food-seeking behavior, at doses up to 20 mg/Kg that did not cause any effect on locomotor activity compared to vehicle treatment (Li et al. 2017). Similar effects were observed using KK-92A in a rat model of alcohol use where KK-92A reduced oral alcohol self-administration and cue-induced reinstatement of alcohol seeking (Maccioni et al. 2021, 2023). The effects of KK-92A on oral alcohol self-administration were observed to show some partial tolerance (Maccioni et al. 2022).

In this study, we did not assess the effect of KK-92A on the animals' locomotor activity as this has been assessed previously. Only high doses of KK-92A (80 mg/kg) affect locomotor activity (Maccioni et al. 2021), which is much higher than the dose used in our study (10 mg/kg). Therefore, we do not expect our results to be influenced by the locomotor effects of KK-92A, especially since behavioral suppression was not observed with the reinstatement of sucrose seeking.

GABA_B_-R is a metabotropic receptor from the G protein-coupled receptor (GPCR) superfamily. It is an obligatory heterodimer receptor that is composed of GABA_B1_ and GABA_B2_ subunits. GABA_B1_ subunits possess the ligand-binding site mediating the interaction between the receptor and the ligand, while GABA_B2_ subunit mediates the downstream signaling via associated G proteins and modulates the affinity of GABA_B1_ subunit to GABA (Qian et al. 2023; Malcangio 2018). These receptors predominantly couple to G_i/o_ proteins, postsynaptically, the effect is inhibitory via activating K^+^ channels (Ulrich and Bettler 2007; Benarroch 2012). Regulators of G-protein signaling (RGS) are one of the main regulators of GABA_B_-Rs (Traynor and Neubig 2005; Balasubramanian et al. 2007) that accelerate the termination of effector stimulation after G protein-coupled receptor activation (Berman et al. 1996). RGS proteins have a prominent role in the activation and deactivation rate of GIRK channels by GPCRs (Doupnik et al. 1997, 2004). In VTA dopamine neurons RGS_2_ modulates the interaction between GABA_B_ receptors and GIRK channels (Labouèbe et al. 2007). In this study, we observed reduced GIRK_2/3_ mRNA levels which could explain the reduced GABA-R mediated currents, but these results will need to be confirmed at the protein expression level in future studies. We didn’t observe any differences in the mRNA expression level of the GABA_B1_ subunit and RGS_2_ in TH^+^ cells of VTA neurons, indicating that the observed reduction in GABA_B_ currents is most probably not attributable to the number of receptors on the surface of the dopamine neurons, nor coupling between GABA_B_-Rs and GIRK channels.

Previous studies have shown that RGS_2_ mRNA level increases in locus coeruleus (LC) neurons 6 h after precipitation of withdrawal in morphine-treated animals, however, it reached the control level after 24 h (Gold et al. 2003). Other studies have also reported that transcriptional changes in RGS following exposure to different stimuli can be rapid (Labouèbe et al. 2007; Taymans et al. 2003). It is possible that while opioids alter RGS_2_ levels, acutely, these normalize during prolonged abstinence. Another study has reported a significant role of RGS6 in VTA dopamine neurons (Spicer et al. 2023), a protein that has a critical role in regulating the GABA_B_-Rs (Garzón et al. 2003). It is possible that other RGS proteins could be affected by opioids, which contributes to reduced coupling between GABA_B_-R and GIRK channels.

GIRK_2/3_ are the predominant GIRK subunits in VTA dopamine neurons (Cruz et al. 2004; Munoz et al. 2016; Labouèbe et al. 2007). In the current study, we found that the mRNA level of GIRK_2_ and GIRK_3_ was decreased in VTA dopamine neurons of rats and mice (only GIRK_2_ was measured in mice) during prolonged abstinence from fentanyl self-administration. Moreover, all TH^+^ neurons expressing the GIRK signal also expressed the GABA_B_ signal. These findings help explain the reduced GABA_B_-R mediated currents reported previously (Moussawi et al. 2020). In another study, it was shown that 24–48 h after chronic methamphetamine injection, GABA_B_-R mediated currents are reduced due to reduced GIRK_3_ levels (Sharpe et al. 2015; Munoz et al. 2016). Other studies have also shown attenuation of GABA_B_-R mediated currents after acute cocaine exposure, but the underlying mechanism was not investigated (Arora et al. 2011). A key point to emphasize that differentiates our study is that all experiments were conducted during prolonged abstinence as opposed to short-term withdrawal, which makes the current results especially salient as a key opioid-induced neuroadaptation mediating long-lasting relapse vulnerability.

D_2_ receptors on midbrain dopamine neurons are activated by dendritic dopamine release resulting in autoregulation of dopamine firing. These D_2_ receptors are coupled to the same GIRK channels as GABA_B_ receptors (Condon et al. 2022). Given the prominent role of D_2_ in regulating the activity of dopamine neurons, we also measured the mRNA expression of D_2_ receptors but did not observe any differences between the fentanyl and control groups. However, as D_2_ receptors activate GIRK channels, and because GIRK mRNA expression decreases during fentanyl abstinence, we predict that D_2_ currents would decrease during prolonged abstinence from fentanyl, which ought to be evaluated in future experiments. Protein expression of GIRKs and other GABA_B_-R signaling mediators were not assessed here. A discrepancy between mRNA levels and protein expression is possible and may reveal significant changes in GABA_B_-R signaling mediators other than GIRKs, which need to be evaluated in future work. Other channels or molecules coupled to GABA_B_ receptors (e.g., calcium channels) could also be affected but warrant further investigation.

An important limitation of the current study is that only changes in mRNA levels were measured. Assessing changes in protein levels is an important next step that was not addressed here. In addition, this study has some limitations regarding the mechanistic link between the reported KK-92A behavioral effects and midbrain dopamine neurons. KK-92A was administered systemically, and therefore, we cannot conclude, from the evidence in this study, that the KK-92A effects were mediated by its actions on GABA_B_ receptors in midbrain dopamine neurons. Further, this study did not assess the physiological effects of KK-92A on GABA_B_ receptor inhibitory post-synaptic currents (IPSCs) in dopamine neurons after fentanyl self-administration.

In summary, our results show decreased GIRK_2/3_ mRNA expression during prolonged abstinence from vapor and IV fentanyl self-administration models in mice and rats, respectively. Our findings also show that the GABA_B_-R PAM, KK-92A, reduces opioid-seeking behavior in rats, possibly by mitigating reduced GABA_B_-R signaling in midbrain dopamine neurons.

## Supplementary Information

Below is the link to the electronic supplementary material.

Supplementary file1 (PDF 1530 KB)

## Data Availability

Data will be made available upon request.
