# Supplementary material for: Reduced GIRK expression in midbrain dopamine neurons during prolonged abstinence from fentanyl self-administration
Source: Psychopharmacology (Berl). 2025 Feb 3;242(7):1653–66. doi: 10.1007/s00213-025-06747-5 (PMC12226691; doi:10.1007/s00213-025-06747-5)
Supplement: Supplementary file 1 — Supplementary file1 (PDF 1530 KB) [file 213_2025_6747_MOESM1_ESM.pdf]

Supplementary materials for

Reduced GIRK expression in midbrain dopamine neurons during prolonged abstinence from fentanyl self-administration

Narges Pachenari[a,b*], Amy L. Channell[a], Andrew J. Belilos[c], Samuel J. Dienel[a], Khaled Moussawi[a,d*]

[a] Department of Psychiatry, University of Pittsburgh, Pittsburgh, PA, USA

[b] Department of Neurobiology, University of Pittsburgh, Pittsburgh, PA, USA

[c] Intramural Research Program, National Institute on Drug Abuse, Baltimore, MD, USA

[d] Department of Neurology, University of California San Francisco, San Francisco, CA, USA

[*]Corresponding authors

Supplementary Materials and Methods

Intravenous (IV) self-administration in rats

Self-administration

Rats were trained to self-administer IV fentanyl on an FR1 schedule for 22 days during which an active lever press resulted in an IV infusion (50 µL) of 3 µg/kg fentanyl over 1.5 seconds with an accompanying 20-second light and tone cues. A 25+/-10 second timeout followed the infusion during which lever presses were counted but were inconsequential. The sucrose group was trained to self-administer sucrose orally on an FR1 schedule. Active lever press was associated with 20-second light and tone cues and resulted in the delivery of 40 µL sucrose (10%) into a well. Sucrose was delivered ~15 seconds after the lever press (to match the delay in the IV drug infusion effect). The sucrose dipper was available for 5 seconds after which it was retracted. Inactive lever presses were recorded but inconsequential. Each training session lasted 3 hours; rats were trained 5 days/week. Each session consisted of three 1-hour blocks, with a maximum of 15 infusions/block to avoid overdose. Sucrose rewards were also capped at 15/block. After 15 infusions, rats went into timeout until the start of the next block.

Catheter surgery

The right external jugular vein was isolated, and a catheter (Instech Laboratories Inc.) was inserted 2.5 cm into the vein, and then anchored with two sutures. The catheter was then tunneled subcutaneously to a port on the back of the rat. Surgeries were performed under isoflurane anesthesia. Surgical areas were shaved and sanitized using betadine and alcohol 70%. On the back, catheter tubing was connected to a port (Instech Laboratories Inc.). After surgery, the catheter was flushed daily with gentamicin for 7 days, and then after 7 days, with heparin and Baytril. Catheter patency was tested with propofol at the beginning and end of the experiment. If the patency test failed at the end of the experimentation, the rat was removed from the study. For sucrose self-administration, rats were implanted with a catheter, but this was not cannulated to the jugular vein.

Apparatus

Operant behavior boxes placed inside noise-reducing cubicles were equipped with two levers, a house light, a ventilator fan, a cue light, a tone generator, a speaker, and either a drug infusion pump or a liquid sucrose delivery system. The two levers were on one side of the box, one active resulting in reward delivery (sucrose vs. fentanyl), and one inactive resulting in no consequences. The system was controlled using Graphic State software and the Coulbourn interface (Harvard Apparatus, USA).

Vapor self-administration in mice

Apparatus

We have previously described vapor self-administration extensively in our papers[22,47]. Operant conditioning was carried out in sealed chambers (14 cm × 20 cm × 23 cm; La Jolla Alcohol Research, La Jolla, CA, USA) positioned within a black Plexiglas enclosure to reduce noise and light interference. Two nose poke holes were installed on opposite sides of the chambers' walls, with white light bulbs positioned above these nose poke holes. A vacuum pump was used to uphold a consistent ambient airflow within the chambers. Drug vaporization was achieved using a vaporizing tank equipped with an atomizer (SMOK TFV8 X-Baby Tank; Shenzhen IVPS Technology, Shenzhen, China), filled with either fentanyl or vehicle solutions. Activation of the atomizer was carried out by an SVS250 vaporizer (Scientific Vapor, OR, USA). For recording nose pokes, controlling the activation of vaporizers, and presenting light cues, we utilized Graphic State software and the Coulbourn interface (Harvard Apparatus, USA). The suction system facilitated the passage of the vaporized drug into the operant chamber upon activation of the vaporizer. The length of time that drug vapor remains in the chamber is contingent upon factors such as airflow rate, the power configuration of the vaporizer, and the duration of vaporization. In our experimental setups, these parameters were fine-tuned to ensure drug clearance within 1 minute following each vapor delivery.

Typically, we maintained an airflow rate of 1 to 2 liters/min, a power setting of 60 W, and a vaporizing duration of 1.5 seconds.

Supplementary Figures:

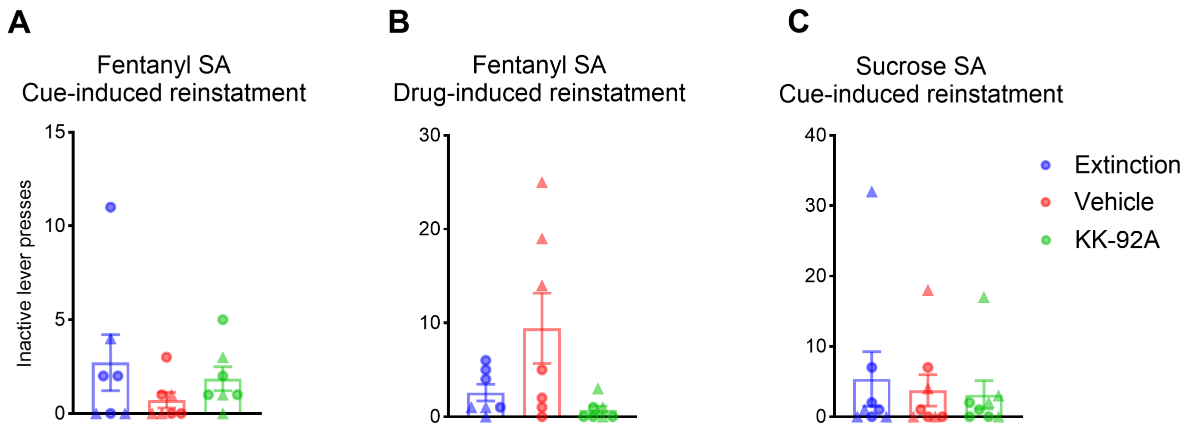

Fig. S1. Inactive lever presses during fentanyl extinction and reinstatement. The number of inactive lever presses during cue- and drug-induced reinstatement in the Fentanyl group and cue-induced reinstatement in the Sucrose group are shown. Triangle and circle data points in bar graphs represent data from female and male animals, respectively.

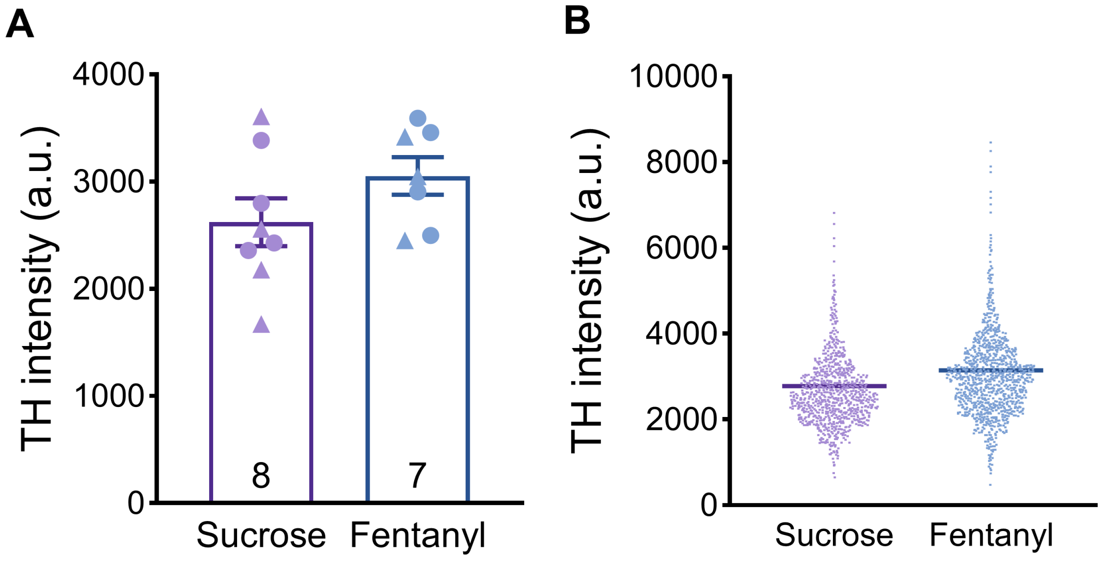

Fig. S2. Tyrosine Hydroxylase (TH) mRNA expression in rats during prolonged abstinence from IV fentanyl self-administration. A) Mean expression of TH mRNA in all cells/ animals was not different between groups (unpaired *t*-test, t_{13}= 1.48, p = 0.16). B) A dot plot of all the data points of TH intensity in each group. a.u. = arbitrary unit. Triangle and circle data points in bar graphs represent data from female and male animals, respectively.

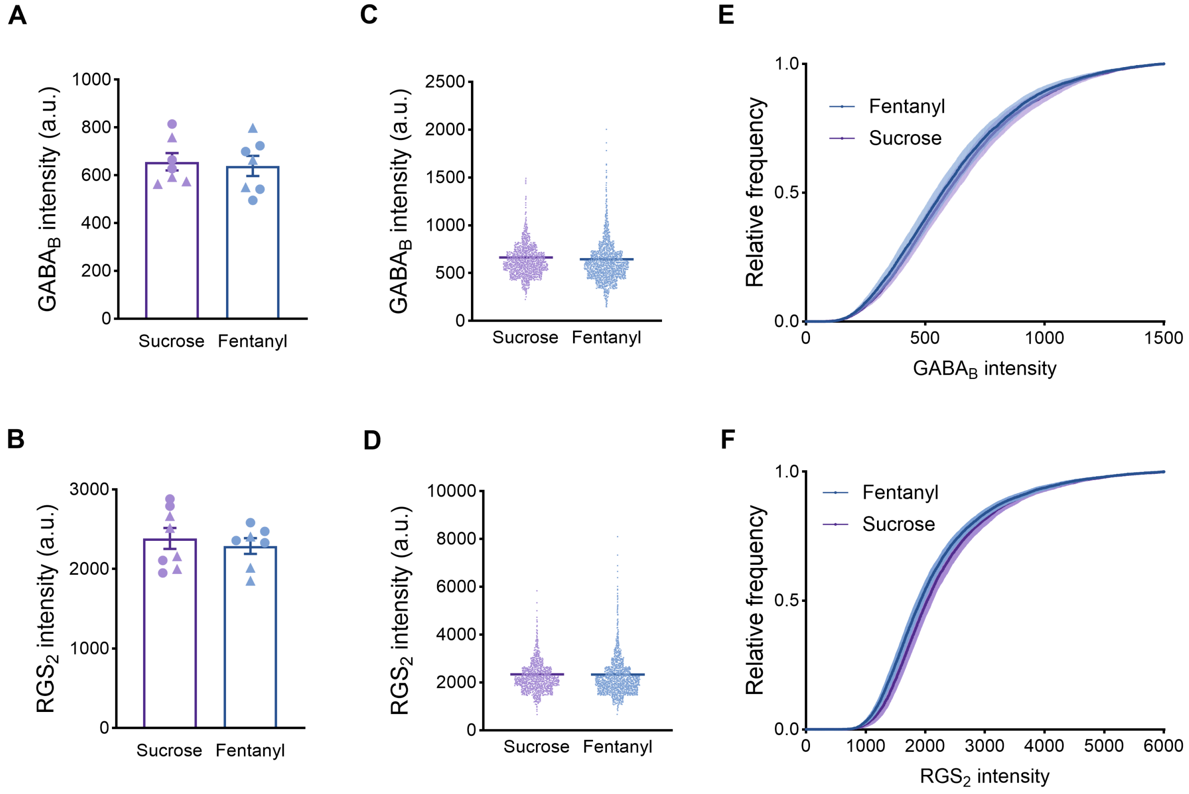

Fig. S3. GABA$_B$ and RGS$_2$ mRNA expression in midbrain dopamine neurons during prolonged abstinence from IV fentanyl self-administration in rats. A and B) Mean expression of GABA$_B$ (A) and RGS$_2$ (B) mRNA in all cells/ animals was not different between groups (unpaired t-test, t_{12}= 0.31, p = 0.76 and t_{13}= 0.56 p = 0.57, respectively). C and D) Dot plots of all the data points of GABA$_B$ (C) and RGS$_2$ (D) mRNA intensity in each group. E and F) Cumulative distribution of all neurons/groups for the GABA$_B$ (E) and RGS$_2$ (F). a.u. = arbitrary unit. Triangle and circle data points in bar graphs represent data from female and male animals, respectively.

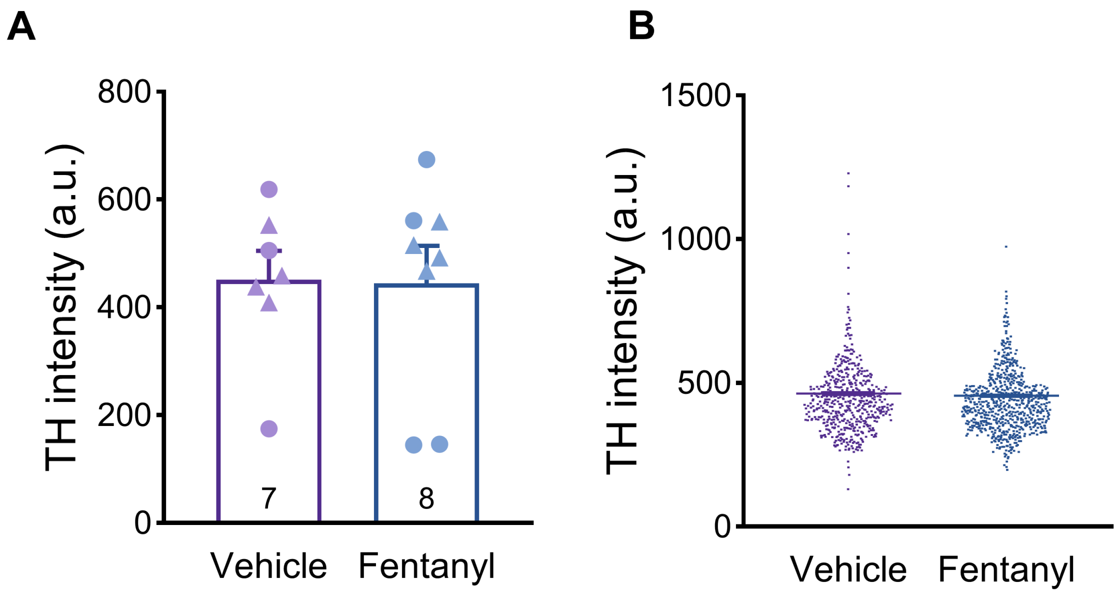

Fig. S4. TH mRNA expression in mice midbrain during prolonged abstinence from vapor fentanyl self-administration. A) Mean expression of TH mRNA in all cells/ animals did not differ between groups (unpaired *t*-test, t_{13}= 0.069, p = 0.94). H) A dot plot of all the data points of TH intensity in each group. a.u. = arbitrary unit. Triangle and circle data points in bar graphs represent data from female and male animals, respectively.

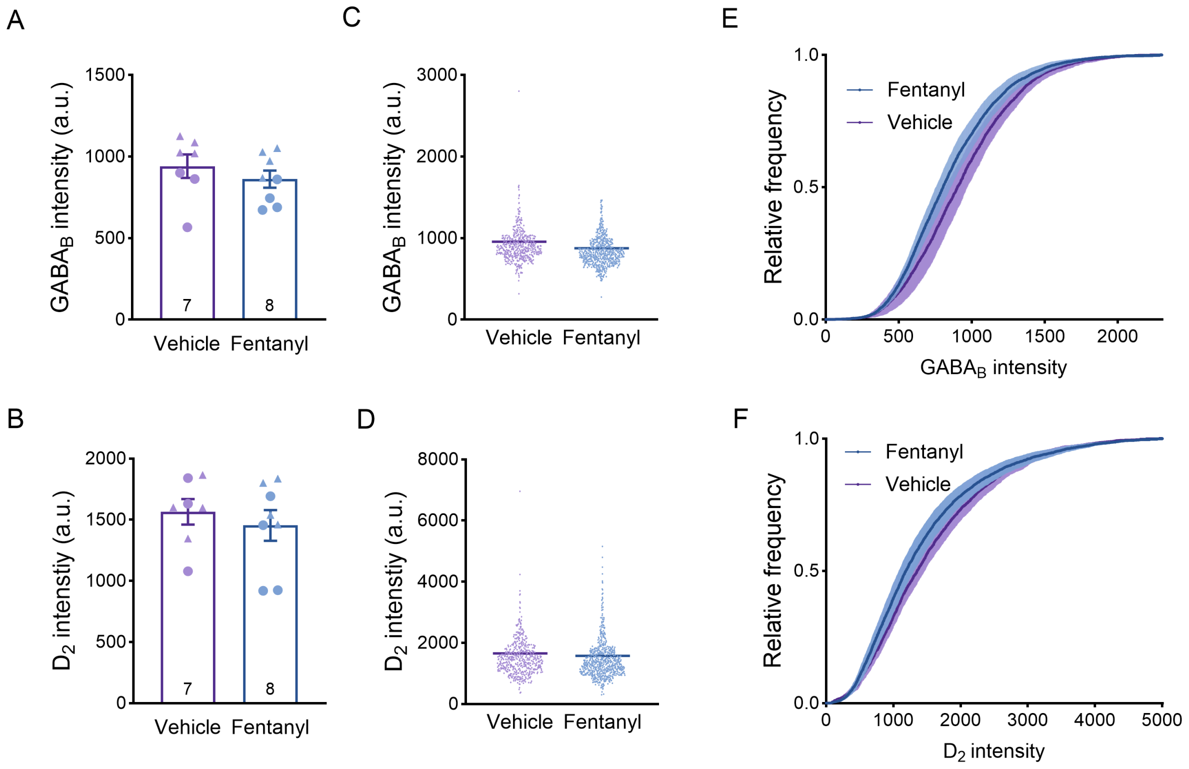

Fig. S5. $GABA_B$ and D_2 mRNA expression in midbrain dopamine neurons during prolonged abstinence from vapor fentanyl self-administration. A and B) Mean expression of $GABA_B$ (A) and D_2 receptor (B) mRNA in all cells/ animals was the same between groups (unpaired t *t*-test, t_{13}= 0.9, p = 0.37 and t_{13}= 0.067, p = 0.51, respectively). C and D) Dot plots of all the data points of $GABA_B$ (C) and D_2 (D) intensity in each group. E and F) Cumulative distribution of all neurons/groups for the $GABA_B$ (E) and D_2 (F). a.u. = arbitrary unit. Triangle and circle data points in bar graphs represent data from female and male animals, respectively.
